# Supplementary material for: Nasal DNA methylation at three CpG sites predicts childhood allergic disease
Source: Nat Commun. 2022 Dec 1;13:7415. doi: 10.1038/s41467-022-35088-6 (PMC9715628; doi:10.1038/s41467-022-35088-6)
Supplement: Supplementary file 1 — Supplementary Information [file 41467_2022_35088_MOESM1_ESM.pdf]

Supplementary Information

Supplementary Figure 1. Sensitivity analysis of PRS

(a)

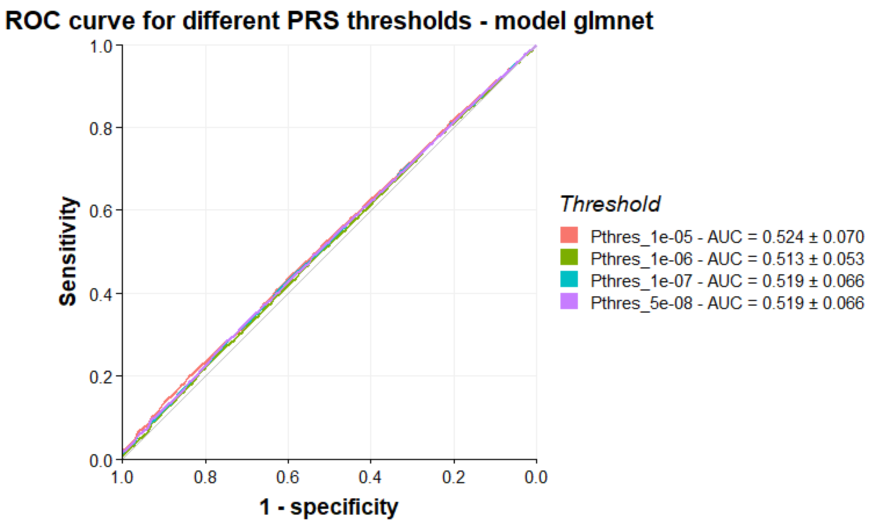

(b)

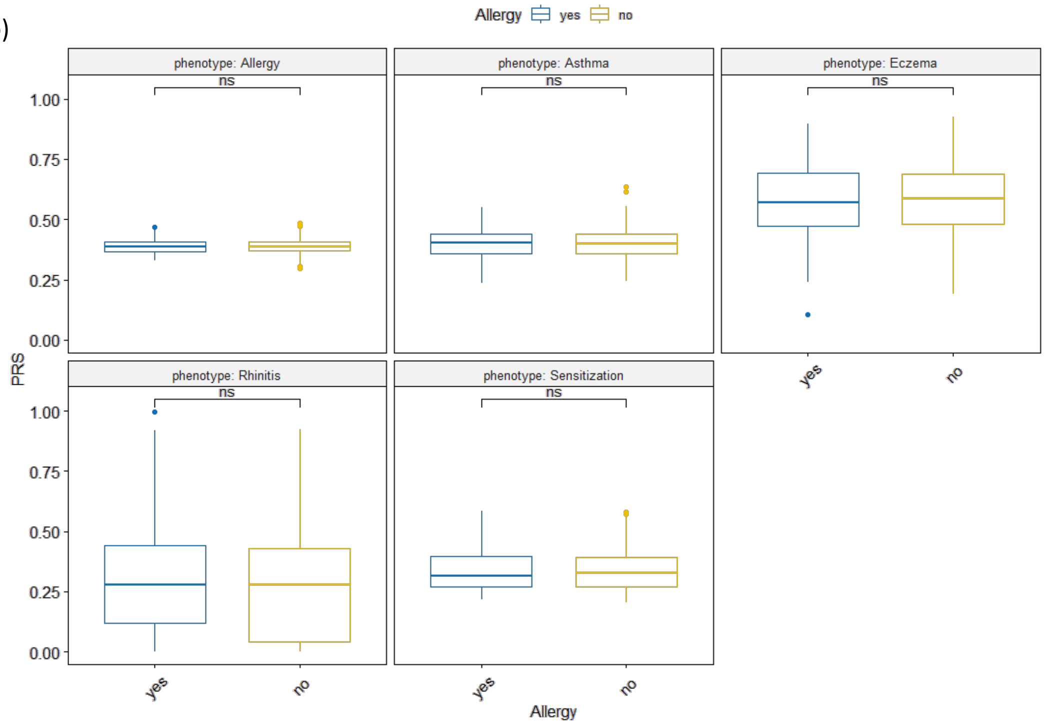

(a) Differential analysis of polygenic risk scores (PRS) using different p-value thresholds. (b) PRS are calculated using matched summary statistics of disease-specific GWAS (allergy, asthma, eczema, rhinitis and IgE sensitization), using a p-value threshold of $1e^{-7}$ for SNP selection. No significant differences in the distribution between case and control groups was observed for the phenotypes (two-sided Student's t-test, p-values from top left to bottom right: 0.99, 0.83, 0.83, 0.61, 0.40, n = 689). Box plots show medians and the first and third quartiles (the 25th and 75th percentiles), respectively. The upper and lower whiskers extend the largest and smallest value no further than 1.5 × IQR. Individual dots below or above the whiskers indicate outliers beyond the 1.5 × IQR range.

Supplementary Figure 2. Precision-recall curves (PRC) for the 3-CpG sites model

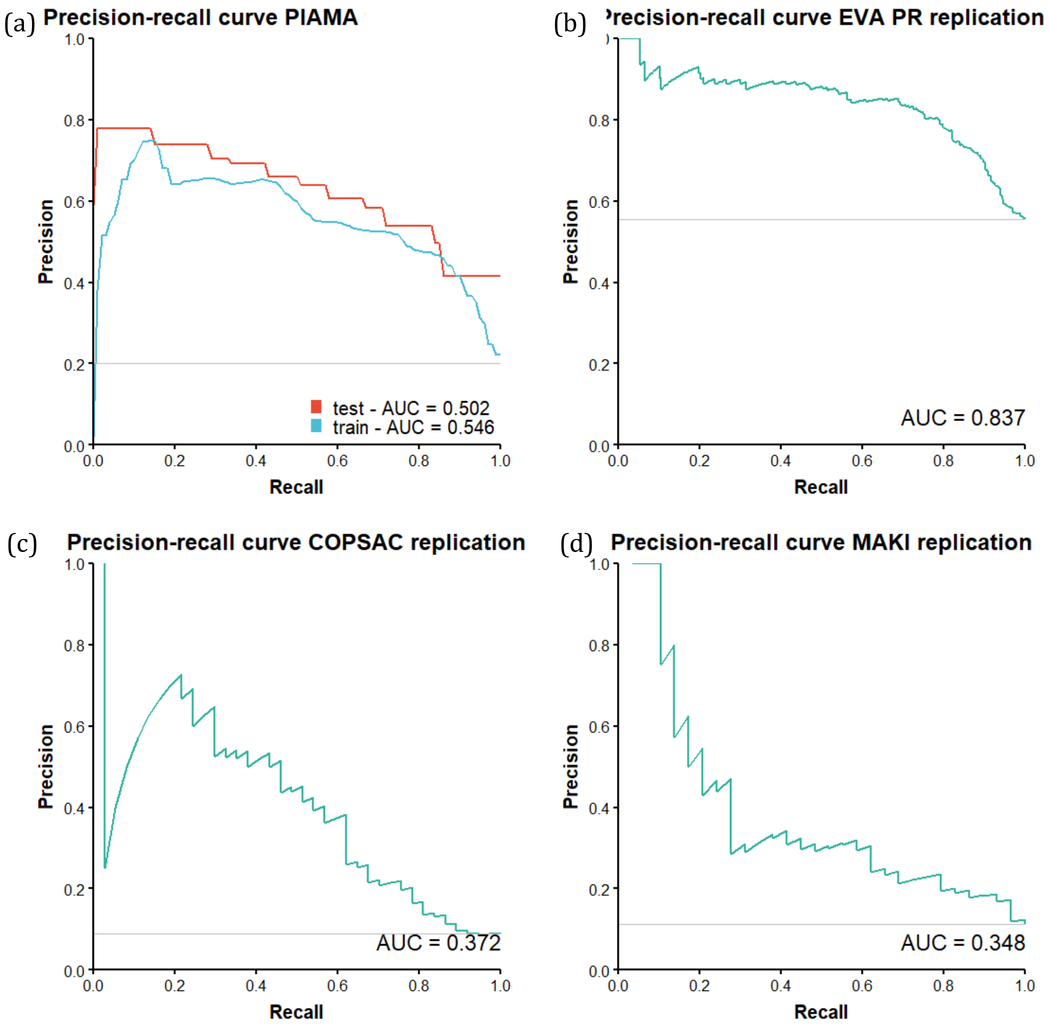

(a) PIAMA discovery cohort; (b) replication cohort EVA-PR; (c) replication cohort COPSAC; (d) replication cohort MAKI

Supplementary Figure 3. Performance curves of Forno et al.'s (2019) prediction panel[21]

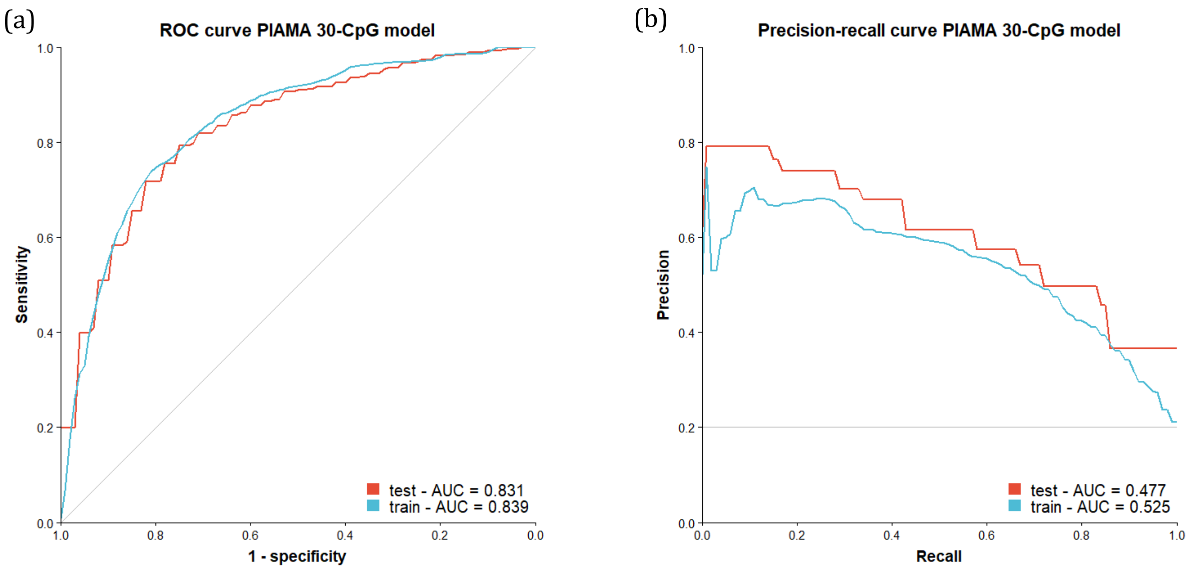

Based on the 30 CpG sites model. These sites are used as model variables for an Elastic Net, which is estimated in the standard 10-times repeated 10-fold cross-validation framework. Their model has been tuned using the same parameters as for the discovery cohort. (a) ROC curve, (b) PRC curve.

Supplementary Figure 4. Age-stratified replication of 3 CpG model in EVA-PR cohort

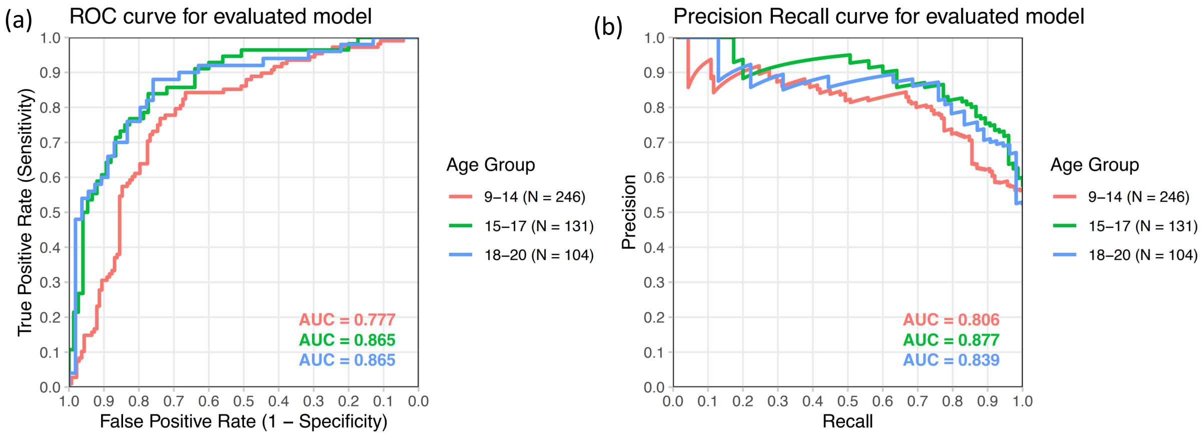

Based on the 3-CpG model that was used for the overall replication, the same model has been used to predict allergic disease within different age groups in the EVA-PR cohort (9-14, 15-17, 18-20) to assess age-dependent performance via ROC (a) and PRC (b).

Supplementary Figure 5. Heatmap of eQTM results

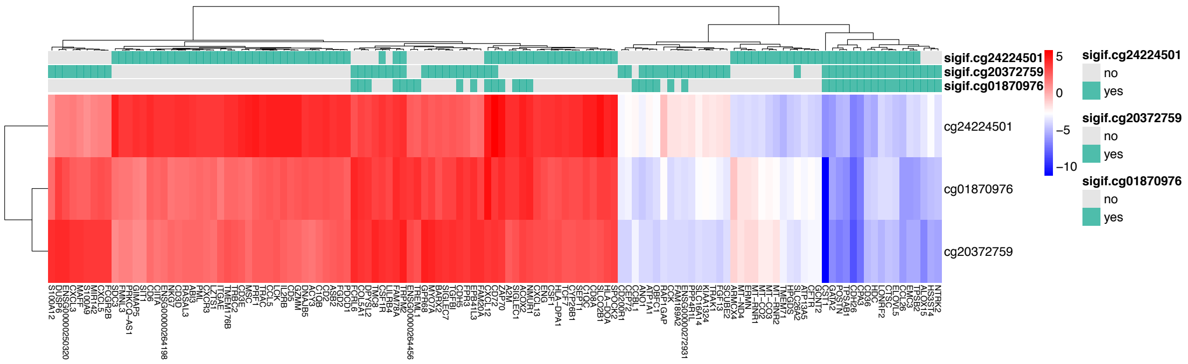

Each column is gene identified by eQTM analysis; on the y-axis are the three CpG sites in the model. Red indicates a positive correlation between a CpG and a gene; blue indicates a negative correlation. The color bars on the top indicate the significance level (yes, FDR<0.05; no, FDR>=0.05) of each gene associated with each CpG site.

Supplementary Figure 6. KEGG pathway enrichment of two gene modules identified by WGCNA

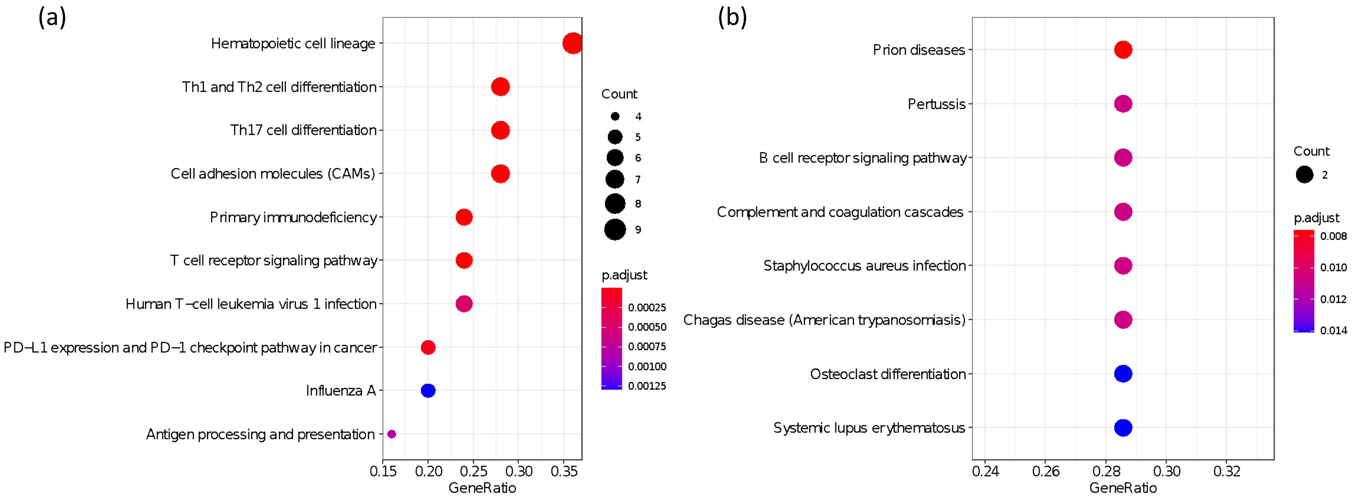

We identified two gene modules from eQTM genes by Weighted Gene Co-expression Network Analysis (WGCNA). The bubble plot shows the results of KEGG pathway enrichment analysis for module 1 (a) and for module 2 (b).

Supplementary Figure 7. Expression patterns of genes associated with three CpG sites in different cell types.

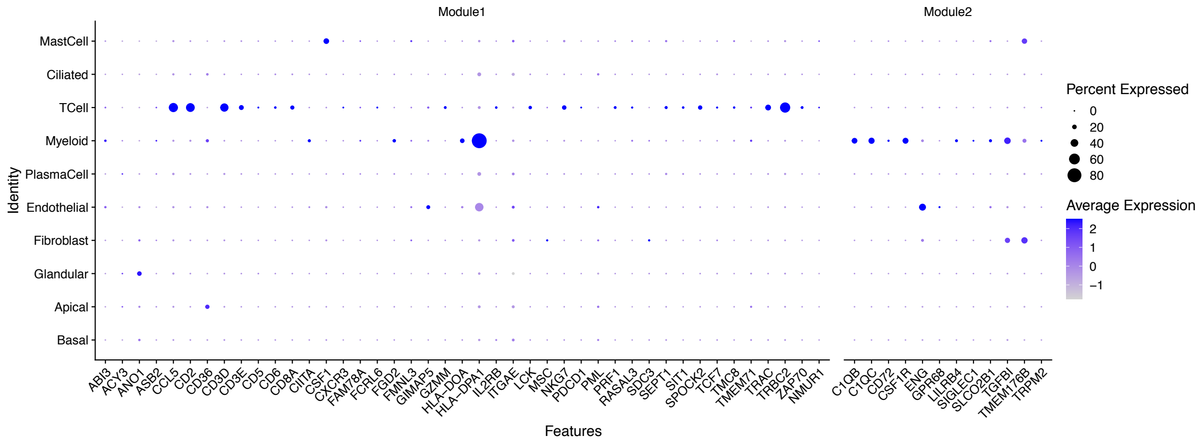

ScRNAseq data were from Ordovas-Montanes et al. (2018), nasal epithelial cells were collected from 6 non-polyp samples and 6 polyp samples[1]. The plot depicts the average expression levels per cell cluster of genes from Module 1 and Module 2 which were available in this scRNA-seq dataset in all cell types.

Supplementary Figure 8. DNA methylation levels of three CpG sites in nasal brushes and blood in the PIAMA birth cohort

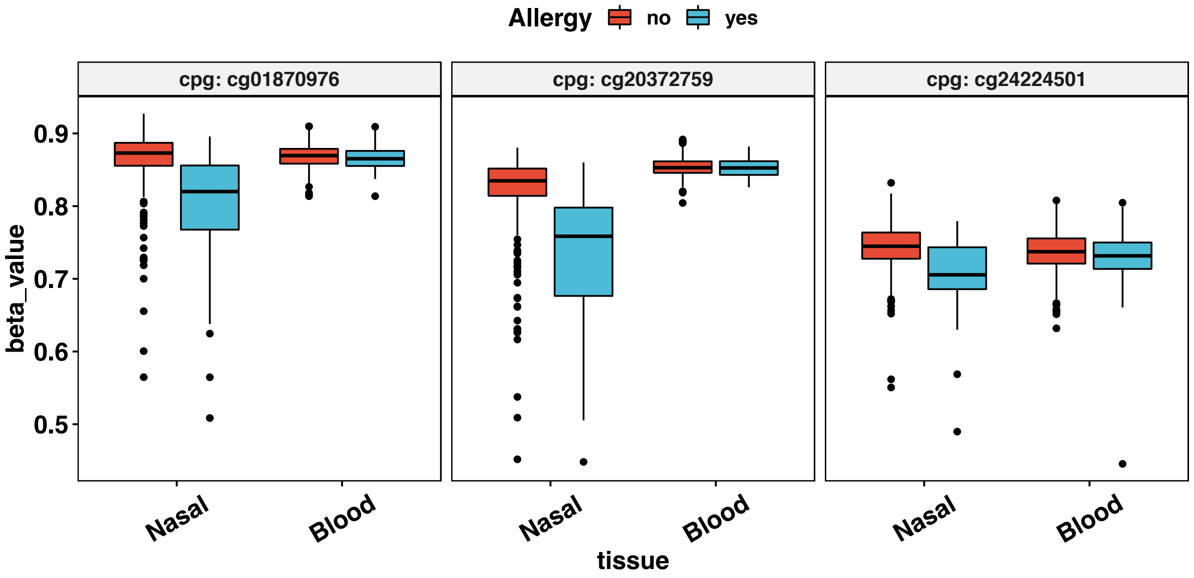

We included 348 samples with matched nasal and blood DNA methylation data, with 67 subjects having allergy and 281 subjects without allergy. Box plots show medians and the first and third quartiles (the 25th and 75th percentiles), respectively. The upper and lower whiskers extend the largest and smallest value no further than 1.5 × IQR, with dots beyond the whiskers indicating outliers (n = 348 samples).

Supplementary Figure 9. Venn diagram of comorbidity

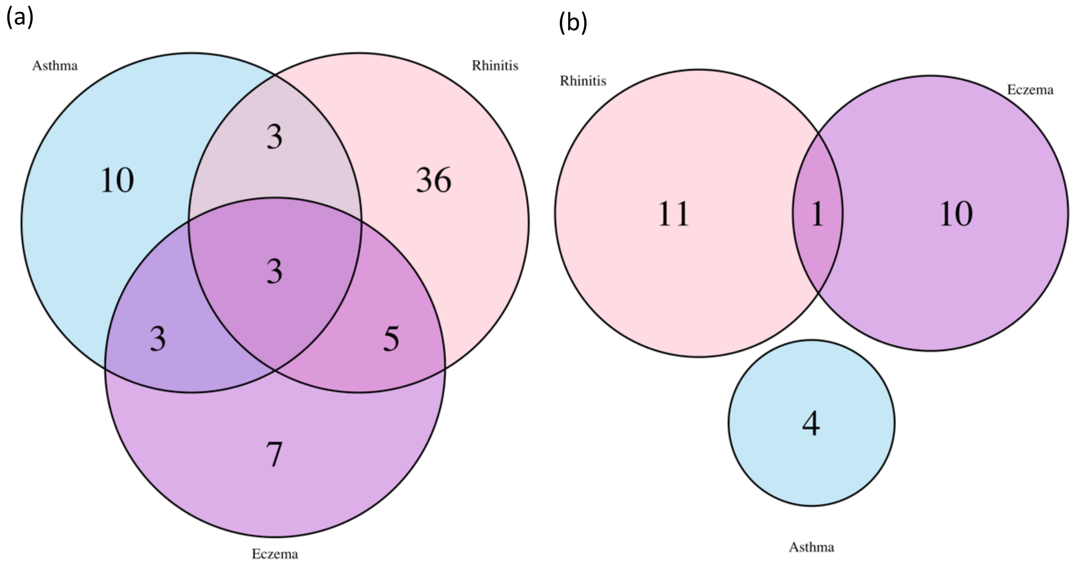

Diagrams show the PIAMA samples with both allergy symptom and IgE sensitization (the allergy phenotype used in this study) (a), and samples with allergy symptom but without IgE sensitization (b).

Supplementary Figure 10. WGCNA analysis of eQTM genes

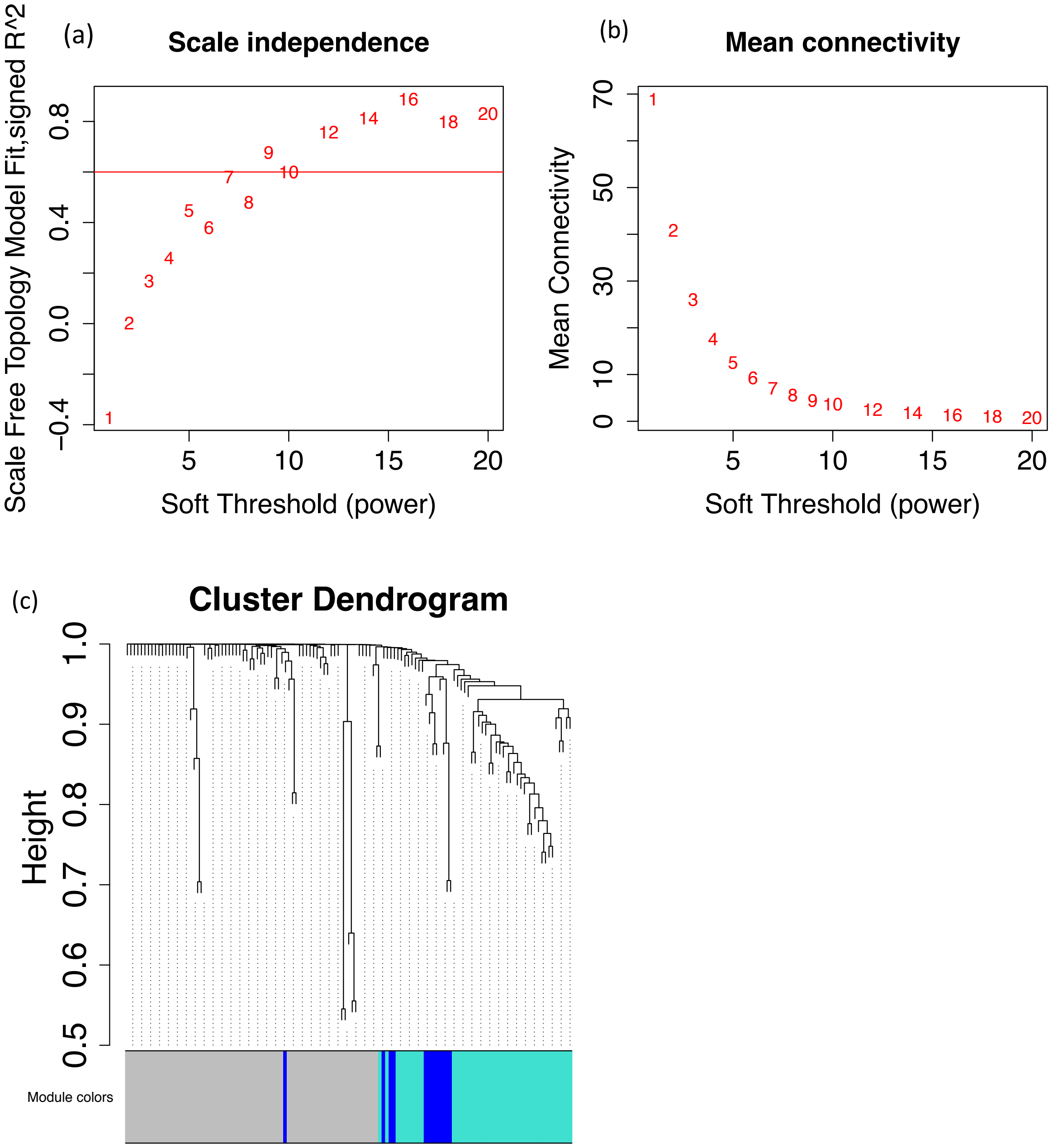

(a-b) Determination of soft-thresholding power in the WGCNA. (a) Analysis of the scale-free fit index for various soft-thresholding powers (β). (b) Analysis of the mean connectivity for various soft-thresholding powers. Soft threshold was selected as 9 and used in the following analysis. (c) Dendrogram of all eQTM genes clustered based on a dissimilarity measure (1-TOM), colors represent identified modules.

Supplementary Methods

Study and population description of discovery cohort

The discovery analysis was performed in the PIAMA birth cohort (Prevention and Incidence of Asthma and Mite Allergy). Details of the cohort have been published previously[2]. In brief, the national study started in 1996 with 3,963 newborns in the Netherlands. Questionnaire-based follow-up of the children took place at 3 months of age, annually from 1 to 8 years of age, and at 11, 14, and 16 years of age, with clinical investigations at ages 4, 8, 12 and 16 years. The Medical Ethical Committees of the participating institutes approved the study (Utrecht and Groningen METC (*Medisch Ethische Toetsings Commissie*) protocol number 12-019/K), and the parents and legal guardians of all participants, and later the participants themselves, gave written informed consent.

In this study, a case with allergic disease is a child with at least one of three allergic diseases (asthma/rhinitis/eczema) and who is specifically IgE positive (>0.35 lU/mL) to any allergen (house dust mite, cat, dactylis (grass) or birch). Asthma was defined as the presence of at least 2 of the following 3 criteria: 1) Doctor diagnosed asthma ever; 2) Wheeze in the last 12 months; and 3) Prescription of asthma medication in the last 12 months. Rhinitis was defined as the presence of sneezing or runny/blocked nose without having a cold in the last 12 months and nose symptoms accompanied by itchy, watering eyes. Eczema was defined as a positive answer to the question: has your child ever had an itchy rash which was coming and going in the last 12 months? If Yes: has this itchy rash affected any of the following places: the folds of the elbows, behind the knees, in front of the ankles, or around the neck, ears or eyes? Serum specific IgE to house dust mite, cat, dactylis (grass) and birch was

measured and classified as positive if ≥ 0.35 IU/ml. Perinatal and environmental factors – including breastfeeding (yes/no), pets during pregnancy (yes/no), maternal smoking (yes/no), elder siblings at home (yes/no), and low birth weight (yes/no) – were assessed by questionnaires that were taken in the first year of life and regularly during follow-up.[1]

The initial dataset included 3,963 participants. The current study used the data of 16-year olds, and included validated questionnaires based on the harmonized MeDALL questionnaire[3], lung function measurements, blood testing for IgE sensitization, and DNA isolation of whole blood cells and nasal brushes. The medical examination at age 16 was restricted to children from the middle and northern regions of the Netherlands (Groningen and Utrecht) due to limited funding. We completed the examinations of in total 802 children at age 16 years. During the examination, blood and nasal samples were collected and used to extract DNA and RNA. In the 802 participants, 348 individuals had complete data of their clinical phenotype at 16 years old, genotype data, blood and nasal DNA methylation data, and environmental exposure data – these were all included in the discovery analysis of model generation (Supplementary Table 8). In the sensitivity analysis of polygenic risk score (PRS), given the possibly limited prediction performance of genetics because of the small sample size, we tried to perform sensitivity analysis using a larger dataset from PIAMA that had both genotype and phenotype data at 16 years old (N = 675), regardless of the availability of other data layers. In the eQTM analysis, we included 244 participants that had both nasal DNA methylation and nasal RNA-seq data available. In the MeQTL analysis, we included 422 participants with both genotype and nasal DNA methylation data.

Data measurement and quality control

Genotype data

Genome-wide genotyping was performed in four phases. Quality control (QC) for each phase was performed and then the data were merged together. The first phase was performed within the framework of the GABRIEL Consortium, using an Illumina Human610 quad array. Genotypes were available from 363 children after QC. A second group of children were genotyped with an Illumina HumanOmniExpress array, with 272 individuals available after QC. A third group of children was genotyped with the Illumina Human Omni Express Exome Array, with 1333 individuals available after QC. A final group of children was genotyped with the Illumina Infinium Global Screening Array, with 107 individuals available after QC. SNPs were harmonized by base pair position annotated to genome build 37. In total, 2075 individuals remained after QC, and data from the four platforms were merged together.

DNA methylation data

We collected samples of peripheral blood, and of nasal epithelial cells by brushing. Briefly, the right nostril of the subjects was examined and the inferior turbinate was located using a speculum and penlight. Brushing was performed with a Cytosoft brush CP-5B (Cyto-Pak) after local anesthesia with 1% lidocaine spray. The lateral area underneath the inferior turbinate was then brushed for 3 seconds and the brush was placed in a 2 ml screw-cap Eppendorf tube and put into a freezer at -80°C until further processing. In total 4 brushes (2 for DNA isolation and 2 for RNA isolation) were collected.

DNA from whole blood was extracted using QIAamp blood kit (Qiagen Benelux BV, Venlo, the Netherlands) and nasal epithelium samples was extracted using DNA investigator kit (Qiagen Benelux BV). DNA concentration was determined by Nanodrop measurement and Picogreen quantification. 500 ng of DNA was bisulfite-converted using the EZ 96-DNA methylation kit (Zymo Research, Irvine, CA, USA), following the manufacturer's standard protocol. After verifying the bisulfite conversion step using Sanger sequencing, DNA concentration was normalized and the samples were randomized to avoid batch effects. One standard DNA sample per chip was included in this step for QC purposes.

Blood and nasal DNA samples were hybridized to the Infinium HumanMethylation450 BeadChip array (Illumina, San Diego, CA, USA). DNA methylation data were pre-processed in R with the Bioconductor package Minfi[4], using the original IDAT files extracted from the HiScanSQ scanner. We implemented sample filtering to remove poor quality samples (call rate <99%). Furthermore, we used the 65 SNP probes to check for concordances between paired DNA brush and blood samples from the same individuals. Paired samples that showed a SNP signal with a Pearson correlation coefficient <0.9 were regarded as sample mix-ups and were excluded from the study. We also verified the methylation distribution of the X-chromosome to verify gender. During processing, the probes on sex chromosomes, the probes that mapped to multiple loci, 65 SNP-probes and the probes containing SNPs at the target CpGs with a MAF >5% were excluded[5]. We implemented "DASEN"[6] to perform signal correction and normalization. After QC, 640 blood samples, 478 nasal samples, and 436,824 probes remained; after matching up with data from all the available layers, 348 samples were used for the analyses.

RNA-sequencing data

Total RNA was extracted using AllPrep DNA/RNA Mini kit (Qiagen Benelux BV). Samples were lysed in 600 µl RLT-plus buffer using an IKA Ultra Turrax T10 homogenizer, and RNA was purified according to the manufacturer's instructions. RNA samples were dissolved in 30 µl RNAse-free water. The concentrations and quality of RNA were checked using a Nanodrop ND-1000 and run on a Labchip GX (PerkinElmer Inc, Waltham, MA, USA).

Initial QC and RNA quantification of the samples was performed by capillary electrophoresis using the LabChip GX (PerkinElmer, Inc). Non-degraded RNA-samples with integrity scores RIN > 6 were selected for subsequent sequencing analysis. Sequence libraries were generated by Poly (A) enrichment using the TruSeq RNA Sample Prep Kit (Illumina) using the Sciclone NGS Liquid Handler (PerkinElmer). In case of contamination of adapter-duplexes, an extra purification of the libraries was performed with the automated agarose gel separation system, Labchip XT (PerkinElmer). Whenever sample preparation failed, either a new sample preparation was attempted or the sample was replaced by a spare nose brush sample from the same individual. The cDNA fragment libraries obtained were loaded in pools of multiple samples into an Illumina HiSeq2500 sequencer using default parameters for paired-end sequencing (2 × 100 bp). If sequencing of a library gave insufficient reads, a second sequencing run was performed to generate additional reads; our target was 15 million read-pairs per sample.

The trimmed fastQ files where aligned to build b37 of the human reference genome using HISAT (version 0.1.5) [7], allowing for 2 mismatches. Reads from separate runs of the same library were merged. Before gene quantification, SAMtools (version 1.2) was used to sort

the aligned reads[8]. The gene level quantification was performed by HTSeq (version 0.6.1p1) using –mode=union --stranded=no and using Ensembl version 75 as the gene annotation database[9].

Quality control metrics were calculated for the raw sequencing data using the FastQC tool (version 0.11.3). Alignments of RNA of 333 subjects were obtained. QC metrics were calculated for the aligned reads using Picard-tools (version 1.130) *CollectRnaSeqMetrics, MarkDuplicates, CollectInsertSize-Metrics and SAMtools flagstat* (http://picard.sourceforge.net). We discarded five samples without replacement due to poor alignment metrics. In addition, we checked for concordance between sex-linked (XIST and Y-chromosomal genes) gene expression and reported sex. Two more samples were discarded for lacking concordance. This resulted in high quality RNA-seq data from 326 subjects.

Expressed features were excluded from analysis if fewer than half the samples had counts per million mapped reads (CPM) of at least 5/M, where M is the median library size in millions. This left 17,156 expressed features for analysis. Raw count data were transformed to log2CPM using *voom* and analyzed in the *limma* package[10].

External replication

We first replicated our model in a cohort of comparable age but different ethnicity (Epigenetic Variation and Childhood Asthma in Puerto Ricans (EVA-PR).

EVA-PR

The Epigenetic Variation and Childhood Asthma in Puerto Ricans (EVA-PR) is a case-control study of asthma in subjects aged 9-20 years; cohort recruitment, procedures, and methods have been described previously[11,12]. Briefly, participants with and without asthma were recruited from randomly selected households in San Juan (PR) from February 2014 through May 2017, using multistage probability sampling. The study was approved by the institutional review boards of the University of Puerto Rico (San Juan, PR) and the University of Pittsburgh (Pittsburgh, PA, USA). Written parental consent and assent from participants <18 years old were obtained, and consent was obtained from participants ≥18 years old. The study protocol included questionnaires on respiratory health, measurement of serum allergen-specific IgE, and taking nasal samples for DNA extraction.

Asthma was defined as physician-diagnosed asthma with at least one episode of wheezing in the previous year. ***Allergic rhinitis*** was defined as hay fever or naso-ocular symptoms (a runny or stuffy nose accompanied by sneezing and itching) apart from a cold or flu in the previous 12 months. ***Eczema*** was defined as a prolonged, itchy, scaly or weepy skin rash in the previous 12 months. ***Atopy*** was defined as ≥1 positive IgE (⩾0.35 IU/mL) to at least one of five common aeroallergens in Puerto Rico: house dust mite, cockroach, cat dander, dog dander, or mouse urinary protein. ***Allergic disease*** was defined as the presence of asthma or allergic rhinitis or eczema AND atopy.

DNA was extracted from nasal specimens collected from the inferior turbinate. Whole-genome methylation assays were performed using HumanMethylation450 BeadChips (Illumina). After QC, 227,836 CpG probes remained. Methylation β-values were calculated as a percentage: $\beta=M/(M+U+\alpha)$, where M and U represent methylated and unmethylated

signal intensities, respectively, and α is an arbitrary offset to stabilize β-values if fluorescent intensities were low. β-values were then transformed to M-values as $\log_2(\beta/(1-\beta))$, and M-values were used in all downstream analyses. Known batch effects (e.g. plates) were removed using an empirical Bayes framework, and *sva* was used to estimate latent factors (LFs) that capture unknown data heterogeneity. To account for population stratification, we also adjusted our models for principal components derived from genotype data (using Illumina HumanOmni2.5 BeadChips).

We also tried to expand the model to other ages especially to two younger cohorts of children aged around 6 years: Copenhagen prospective studies on asthma in childhood (COPSAC)[13] and the Dutch MAKI trial[14].

COPSAC

The $COPSAC_{2010}$ cohort is an ongoing, prospective mother-child cohort comprising 731 children born to unselected mothers from Zealand, Denmark, in 2009-2010 as described previously[13]. At week 24 of pregnancy, women were randomly assigned to receive n-3 long chain polyunsaturated fatty acids (n = 362) and placebo (n = 369). Baseline information as well as details of this randomized controlled trial have been published[15].

The study was conducted in accordance with the guiding principles of the Declaration of Helsinki and was approved by the Local Ethics Committee (COPSAC2010: H-B-2008-093), and the Danish Data Protection Agency ($COPSAC_{2010}$: 2015-41-3696). Both parents provided written informed consent before enrolment.

Asthma, Eczema and Rhinitis were diagnosed longitudinally by research physicians following the same standardized algorithm. An asthma diagnosis required a certain burden of troublesome lung symptoms, response to treatment with inhaled corticosteroids and relapse after withdrawal of treatment, which was reported previously[15]. In this replication, current **asthma** by age 6 years was defined as still having asthma symptoms and needing regular treatment with inhaled corticosteroids in the previous year.

Rhinitis is defined as the presence of sneezing, runny, itchy or stuffed nose and the absence of infection. Specifically, besides absence of infection, two or more of the following symptoms experienced in more than one hour for more than two days: Runny nose, sneezing, itchy or blocked nose. If these criteria are not met, but the symptoms persist for more than one season or are relieved by antihistamines we include them as cases. Rhinitis symptoms were evaluated at 6 years of age.

Eczema is defined as positive according to the definitions in Hanifin & Rajka. We required three major and three minor features to be regarded as a case[16]. Eczema symptoms were evaluated at 6 years of age.

Sensitization (sIgE) is defined as specific IgE against the tested allergen (>0.35 lU/mL) at age six. We used inhalants allergens to define sensitization, and we measured house dust mite, cat epithelium and dander, dog dander, timothy grass, common silver birch, mugwort, general test for molds, *Cladosporium herbarium*, *Aspergillus fumigatus*, *Alternaria tenuis* and tissue transglutaminase. A child was considered sensitized if it had an inhalant sensitization.

For the current study, we used the MeDALL **allergy** definition as was proposed by experts[3]. A child was considered to have allergy if he/she had at least one of three diseases: asthma/rhinitis/eczema, as well as inhalant sensitization IgE (>0.35 lU/mL).

RNA was collected from inferior turbinate epithelial cell scrapings from 562 children. Samples were obtained using a rhino-probe nasal curette, stored in RNAprotect Cell Reagent (Qiagen, Germantown, MD, USA), and then cryopreserved at −80°C until nucleotide extractions could be performed[17]. After removing samples that did not pass QC or without genotype information (n = 59), 503 samples remained.

Out of 866,836 probes on the Illumina Infinium Methylation EPIC array, we removed 23,172 with a detection p-value of > 0.01 in 90% of the samples, 18,695 located on sex chromosomes, and 120,903 probes that overlapped a known common SNP (MAF > 0.05) or mapped to multiple positions as a result of cross-hybridization. 703,565 CpG sites passed QC and were used in downstream analyses. Normalization was performed using the SWAN algorithm from the R package *minfi* (version 1.26) and quantile normalization from the R package *lumi* (version 2.36). Principal component analysis (PCA) was used to identify the effect of confounding variables on DNA methylation: DNA concentration, slide and site of sampling significantly correlated with at least one of the first ten principal components and were included in the final model.

MAKI

The MAKI trial enrolled 429 otherwise healthy, late preterm infants between 2008 and 2010; they were born at 33 to 35 weeks gestation. Infants were randomly assigned to receive palivizumab (n = 214) or placebo (n = 215) during their first RSV season. Baseline characteristics at randomization are available at NEJM.org[14]. Details about the design,

definitions, protocol of the primary study, follow-up study and clinical assessment have been previously described[14,18].

Information on asthma, rhinitis, eczema and IgE sensitization was obtained from clinical assessment at age 6 years. **Asthma** was defined as parent-reported wheeze or asthma medication usage and physician diagnosed asthma. **Rhinitis** was defined as the child having suffered from sneezing, a runny nose, or a blocked nose in the past 12 months while he / she did not have a cold. **Eczema** was defined as the child ever having suffered from itchy skin rash on the knee cavities/ the front of the ankles/ the neck/ inside of the elbows/ around the eyes or ears, in the last 12 months. IgE sensitization was defined as specific IgE to inhalant allergens at age 6 years. **Allergic disease** was defined as the presence of asthma or rhinitis or eczema AND IgE sensitization (>0.35 IU/ml).

Nasal epithelial cells were collected by brushing around age 6. Briefly, the right nostril of the subjects was examined and the inferior turbinate was located using a speculum and penlight. The lateral area underneath the inferior turbinate was then brushed for 3 seconds with two brushes (Copan, 56380CS01 FLOQswabs) and these were placed in a 2 ml screw-cap Eppendorf tube and put into a freezer at – 80o C until further processing.

DNA was extracted from nasal brushes using the DNA investigator kit (Qiagen, Benelux BV, Venlo, the Netherlands). This was followed by precipitation-based purification and concentration using GlycoBlue (Ambion). 500 ng of DNA was bisulfite-converted using the EZ 96-DNA methylation kit (Zymo Research), following the manufacturer’s standard protocol. After verification of the bisulfite conversion step using Sanger Sequencing, DNA concentration was normalized and the samples were randomized to avoid batch effects.

One standard DNA sample per chip was included in this step for quality control. Study personnel and technicians were blinded for the intervention.

In total, 296 nasal epithelium samples with sufficient DNA quality and quantity were hybridized to the Infinium HumanMethylationEPIC BeadChip array (Illumina, San Diego, CA, USA). DNA methylation data were pre-processed in R (version 3.3.2) with the Bioconductor package *Minfi*, using the original IDAT files extracted from the HiScanSQ scanner. We implemented sample filtering to remove 6 bad quality samples (call rate <99%) and 16 samples with gender mismatch. During processing, bad quality probes which failed more than 10% of the samples, the probes on sex chromosomes, the probes that mapped to multiple loci, and the probes containing SNPs at the target CpG sites with a MAF>5% in European populations were excluded. We subsequently implemented stratified quantile normalization. After quality control, 274 (63.8%) of the collected samples and 790,437 probes remained for further analyses.

Nasal single cell RNA-seq cohort

Nasal brush samples from 4 asthma patients and 5 healthy controls were collected from inferior turbinate by Cytosoft brush CP-5B (Cyto-Pak). The brushes were collected in a 50mL tube containing HBSS(Lonza) + 1%Pen/Strep. Cells were spinned down at 560xg for 5 min. Cell pellet was then resuspended in HBSS containing 1mg/ml Collagenase D and 0.1mg/ml DNase I (Roche) and placed at 37°C for 1 hour with gentle agitation. Cell suspension was pushed through a 70uM nylon cell strainer (Falcon) and spinned down at 560xg for 5 min. Next, cells were washed with PBS containing 1% BSA (Sigma Aldrich). Single cell suspension was cleared of red blood cells using a Red Blood cell lysis buffer (eBioscience).

Cell suspension was counted manually using a haemocytometer and concentration was adjusted to a minimum of 300 cells/ul. Cells were loaded according to the standard protocol of Chromium single cell 3'kit. Following steps were performed according to Single Cell 3'Reagent Kits V2 User guide. We performed RNA-sequencing on Illumina Hiseq 4000 or NOVA-Seq 6000 aiming to achieve a mean coverage of 50k -100k reads/cell. 10X Genomics raw sequencing data was processed using CellRanger software and the 10X human genome GRCh38 1.2.0 release as the reference. Downstream analyses including clustering of cells and identifying cluster marker genes were performed using the R software package Seurat version 4 (https://github.com/satijalab/seurat).

Mediation analysis

To evaluate what proportion, if any, of an association between SNP and asthma was mediated through changes in the DNA methylation level of the three CpG sites, we conducted mediation testing using R package mediation[19]. The SNP list (N=75) included in this analysis was obtained from previous GWAS of allergic disease[20]. We performed an MeQTL analysis that associated the three CpG sites with SNPs previously associated with allergic disease. For the most significant SNP- CpG pair, we performed the mediation analysis. First, we tested the total effect of SNP on allergic disease (model: Allergic disease ~ SNP). Second, we tested the effect of the independent variable (SNP) on the mediator (DNA methylation; model: DNAm ~ SNP). Third, we tested the mediator's (DNA methylation) and independent variable's (SNP) effect on the dependent variable (allergic disease; model: Allergic disease ~ SNP +DNAm). Lastly, we compared the direct and the indirect effect and estimated the mediation effect.

WGCNA

We then performed WGCNA[21] on the genes identified by eQTM analysis. In brief, WGCNA determines the modules according to weighted co-expression of the genes. The first step is the construction of co-expression network. By raising the absolute value of the correlation to a power $\beta \geqslant 1$ (soft thresholding), the weighted gene co-expression network construction emphasizes high correlations at the expense of low correlations. Here, modules are clusters of genes with high absolute correlations. The detection of the module is the step after the construction of co-expression network, WGCNA identified modules using unsupervised hierarchical clustering. We set the minimum size of the module as 8 genes, and soft threshold β as 9 (according to the analysis of the scaled-free fit index for various soft-thresholding powers, see Supplementary Figure 10).

Supplementary Table 1. Comparison of prediction performance for different machine learning techniques

	Training				Testing			
Model	ROC AUC mean	SD	PRC AUC mean	SD	ROC AUC mean	SD	PRC AUC mean	SD
Elastic Net	0.874	0.009	0.560	0.020	0.835	0.079	0.470	0.126
Naive Bayes	0.894	0.179	0.693	0.266	0.720	0.132	0.345	0.143
Neural Net (1-layer)	0.931	0.037	0.733	0.100	0.730	0.099	0.383	0.115
Random Forest	1.000	0.000	0.983	0.000	0.838	0.076	0.464	0.130
Support Vector Machine (Radial kernel)	0.904	0.013	0.636	0.026	0.799	0.090	0.443	0.128
XGBoost	0.988	0.013	0.943	0.041	0.826	0.077	0.464	0.127

Caption

ROC AUC: receiver operating characteristic, area under curve

PRC AUC: precision-recall curve, area under curve

Supplementary Table 2. Summary of ranking of features from different data layers

		Feature ranking			
Daya layer	Full feature model (No.	No. in top 150 (% of top X)	No. in top 100 (% of top X)	No. in top 50 (% of top X)	No. in top 10 (% of top X)
Host	2	1 (0.7%)	0 (0%)	0 (0%)	0 (0%)
Environment	4	2 (1.3%)	0 (0%)	0 (0%)	0 (0%)
Perinatal	2	0 (0%)	0 (0%)	0 (0%)	0 (0%)
Genetics	106	3 (2%)	1 (1%)	0 (0%)	0 (0%)
Blood DNA methylation	219	76 (50.7%)	43 (43%)	7 (14%)	0 (0%)
Nasal DNA methylation	134	68 (45.3%)	56 (56%)	43 (86%)	10 (100%)

Caption

Host: age and gender

Environment: cats or dogs during pregnancy, maternal smoking (mother smoking in pregnancy but stopped between 12 and 16 weeks and mother smoking in pregnancy at 16 weeks), older siblings at home

Perinatal: low birth weight and breastfeeding

Genetics: allergy SNP dosages and polygenic risk scores (PRS) for the combined allergy phenotype, as well as for asthma, rhinitis, eczema and IgE-sensitization

Blood methylation: 219 CpG sites from blood cells that were previously associated with allergy

Nasal methylation: 134 CpG sites from nasal cells that were previously associated with allergy

Supplementary Table 3. Rank products of top-10 ranked features

Rank	Feature	Rank product	AUROC
1	methylNasal - cg20372759	2963.495	0.816
2	methylNasal - cg01870976	1717.827	0.824
3	methylNasal - cg24224501	1541.129	0.856
4	methylNasal - cg22862094	1454.589	0.854
5	methylNasal - cg16027132	1441.167	0.848
6	methylNasal - cg20790648	1218.264	0.855
7	methylNasal - cg24707200	1070.770	0.852
8	methylNasal - cg15006973	921.767	0.854
9	methylNasal - cg08844313	917.173	0.855
10	methylNasal - cg22689016	714.923	0.850

Caption

Rank product statistic[22] is computed based on 10 times repeated 10-fold cross-validation, as described in Methods. AUROC refers to the AUC of the ROC curve.

Supplementary Table 4. Significance testing of number of model features

		Model with j features									
		1	2	3	4	5	6	7	8	9	10
Model with i features	1	-	0.555	####	0.062	0.095	0.103	0.126	0.110	0.115	0.191
	2		-	####	0.065	0.120	0.102	0.112	0.092	0.104	0.191
	3			-	0.759	0.302	0.953	0.812	0.880	0.995	0.702
	4				-	0.281	0.924	0.919	0.999	0.892	0.791
	5					-	0.556	0.696	0.611	0.571	0.885
	6						-	0.733	0.876	0.922	0.553
	7							-	0.719	0.652	0.697
	8								-	0.801	0.608
	9									-	0.291
	10										-

Caption

Table displays the p-values of the performance difference between model with i features compared to a model with j features.
These results show that from 1 feature to 3 is a significant improvement (alpha=.05), as well as the step from 2 to 3 features.
Above 3 features, there is no significant improvement. Test statistic based on two-sided corrected repeated k-fold cv test.

Supplementary Table 5. Annotation of three CpG sites in our parsimonious model

CpG	CHR	BP	GREAT annotation
cg20372759	12	58162287	METTL21B(-4095),CYP27B1(-1254)
cg01870976	15	101887154	SNRPA1(-51699),PCSK6(+142718)
cg24224501	7	102566723	ARMC10(-148604),LRRC17(+13286)

Supplementary Table 6. Three-CpG model specification

Feature name	Coefficient
(Intercept)	-0.0025882
methylNasal - cg20372759	0.13323378
methylNasal - cg01870976	0.11658298
methylNasal - cg24224501	0.09059412

Caption

Coefficients for three-CpG model of Elastic Net model, resulting from hyperparameter optimization on PIAMA cohort using the *caret* package in R (alpha=0.1, lambda=0.9)

Supplementary Table 7. Performance metrics for all four cohorts

	PIAMA	EVA_PR	COPSAC	MAKI
Accuracy	0.815	0.771	0.921	0.892
Sensitivity	0.638	0.787	0.270	0.034
Specificity	0.857	0.752	0.984	1.000
Pos Pred Value	0.533	0.798	0.625	1.000
Neg Pred Value	0.910	0.739	0.933	0.891
Precision	0.533	0.798	0.625	1.000
Recall	0.638	0.787	0.270	0.034
F1	0.569	0.792	0.377	0.067

Caption

All metrics are based on the 3-CpG model. Metrics for PIAMA are from the test performance.

Supplementary Table 8. Gene modules identified from eQTM genes by WGCNA

Module1	Module2
ZAP70	SIGLEC1
FAM78A	CD72
ANO1	TRPM2
CD36	GPR68
NMUR1	TGFBI
FCRL6	CSF1R
TMC8	LILRB4
CD6	TMEM176B
TCF7	ENG
ITGAE	SLCO2B1
IL2RB	C1QC
ASB2	C1QB
RASAL3	
NKG7	
SPOCK2	
ABI3	
CD5	
CD2	
ACY3	
SIT1	
PML	
FGD2	
CD8A	
CCL5	
FMNL3	
SDC3	
TMEM71	
CD3D	
MSC	
CIITA	
SEPT1	
PRF1	
LCK	
CSF1	
CXCR3	
PDCD1	
GIMAP5	
GZMM	
CD3E	
HLA-DOA	
TRBC2	
TRAC	
HLA-DPA1	
ENSG00000264198	

Supplementary Table 9. Sample characteristics of single-cell RNASeq dataset

		N
Total		9
Age		51.4 ± 8.4
Gender male (%)		6 (66.7%)
BMI		28.1 ± 4.9
Smoking (%)		2 (22.2%)
Disease		
	Asthma (%)	4 (44.4%)
	Rhinitis (%)	3 (33.3%)
	Eczema (%)	3 (33.3%)

Supplementary Table 10. MeQTL analysis of three CpG sites with allergy-related SNPs identified by Ferreira et al. (2017)

SNP ID	CHROM	effect_allele	other_allele	risk allele of allergic disease	POS	Gene	CpG ID	beta	se	pvalue
rs9372120	6	G	T	G	106667535	[ATG5]	cg20372759	-0.203	0.060	8.61E-04
rs144829310	9	T	G	T	6208030	RANBP6--[]-IL33	cg24224501	0.068	0.026	9.28E-03
rs7130753	11	T	C	C	111470567	LAYN-[]-SIK2	cg24224501	0.049	0.021	0.019
rs9372120	6	G	T	G	106667535	[ATG5]	cg01870976	-0.121	0.052	0.022
rs3749833	5	C	T	C	131799626	[C5orf56]	cg01870976	-0.091	0.039	0.022
rs76167968	1	C	T	T	35681738	SFPQ-[]-ZMYM4	cg24224501	0.082	0.036	0.023
rs2104047	14	C	T	T	68754417	[RAD51B]	cg01870976	-0.087	0.038	0.023
rs10486391	7	G	A	A	20376018	[ITGB8]	cg20372759	-0.084	0.037	0.024
rs4574025	18	T	C	T	60009814	[TNFRSF11A]	cg20372759	-0.087	0.039	0.027
rs56129466	11	G	A	A	128158189	KIRREL3-AS3---[]--ETS1	cg24224501	-0.047	0.022	0.031
rs11204896	1	G	C	C	151796742	[RORC]	cg01870976	0.125	0.058	0.032
rs6869502	5	T	A	T	110166083	SLC25A46-[]--TSLP	cg20372759	-0.119	0.058	0.043
rs4848612	2	A	G	A	112388538	BCL2L11--[]--ANAPC1	cg24224501	-0.039	0.019	0.043
rs3749833	5	C	T	C	131799626	[C5orf56]	cg20372759	-0.091	0.046	0.047
rs5029937	6	T	G	G	138195151	[TNFAIP3]	cg24224501	-0.101	0.051	0.047

Caption

Gene: the nearest genes of the SNP

Linear regression model was used to perform the MeQTL analysis

Supplementary Table 11. Mediation analysis of SNP (rs9372120) - DNA methylation (cg20372759) - allergic disease

Model	variable tested	Estmate	SE	t value	P value
1. Allergic disease ~ SNP	SNP	0.099	0.043	2.297	0.022
2. DNAm ~ SNP	SNP	-0.229	0.06	-3.849	0.00013
3. Allergic disease ~ SNP +DNAm	SNP	0.024	0.039	0.615	0.539
Mediation analysis		Estimate	95% CI Lower	95% CI Upper	P value
	ACME	0.075	0.036	0.12	<2E-16
	ADE	0.024	-0.058	0.11	0.554
	Total Effect	0.099	0.016	0.19	0.018
	Proportion Mediated	0.757	0.327	3.49	0.018

Caption

ACME: Average Causal Mediation Effects

ADE: Average Direct Effects

Linear regresssion was used in each single association analysis

Supplementary Table 12. Sample characteristics of 16-year follow-up on subjects (included/ not included in the analyses) and subjects who did not participate in the 16-year follow-up medical examination

		Subjects who participated in 16-year follow-up medical examination		Subjects who did not participate in the 16-year follow-up medical examination (n=3161)
		Subjects in the analyses (n=348)	Subjects not in the analyses (n=454)	
General information	Age (years)	16.3 ± 0.2	16.4 ± 0.2	NA
	Sex: number of males	169 (48.6%)	217 (47.8%)	1668 (52.8%) *
Baseline information	Atopic mother	122/ 348 (35.1%)	135/ 454 (29.7%)	980/ 3161 (31.0%)
	Atopic father	112/ 348 (32.2%)	154/ 453 (34.0%)	951/ 3156 (30.1%)
	Breast feeding	305/ 348 (87.6%)	400/ 454 (88.1%)	2495/ 3094 (80.6%)*
	Maternal education			*
	Low	56/ 348 (16.1%)	65/ 454 (14.3%)	773/ 3005 (25.7%)
	Medium	135/ 348 (38.8%)	181/ 454 (39.9%)	1266/ 3005 (42.1%)
	High	157/ 348 (45.1%)	208/ 454 (45.8%)	966/ 3005 (32.1%)
	Maternal smoking	54/ 348 (15.5%)	51/ 447 (11.4%)	598/ 3105 (19.3%) *
	Birth weight (g)	3564.4 ± 533.9	3547.5 ± 507.9	3495.0 ± 552.3 *
Information of 16 years follow-up	Asthma	23/ 348 (6.6%)	43/ 418 (10.3 %)	
	Rhinitis	59/ 348 (17.0%)	74/ 409 (18.1%)	
	Eczema	29/ 348 (8.3%)	**56/ 419 (13.4%)**	
	Positive allergen-specific IgE	162/ 348 (46.6%)	182/ 373 (48.8%)	
	Allergy	67/ 348 (19.3%)	93/ 407 (22.9%)	
	Pets	183/ 291 (62.9%)	**206/ 375 (54.9%)**	
	Current smoking	37/ 297 (12.5%)	47/ 356 (13.2%)	

Caption

Breast feeding: includes any breast feeding

Maternal smoking: maternal smoking during at least the first 4 weeks of pregnancy

Continuous variables are presented as mean ± SD; categorical variables are presented as number of ("yes")/ (total number (non-missing)) (percentage)

Bold: significant difference compared to subjects in the analyses (P<0.05), t test for continous variable and chi-square test for categorical variable

* significant difference compared to subjects who participated in the 16-year follow-up medical examination (P<0.05), t test for continous variable and chi-square test for categorical variable

Supplementary Table 13. Literature source of initial feature selection

Daya layer	Study reference	Number of features selected (post QC)
Environment	Miliku and Azad (2018)	-
Perinatal	Murrison *et al.* (2019)	-
Genetics SNPs	Ferreira *et al.* (2017)	75
Genetics PRS - Asthma	Demenais *et al.* (2018)	660
Genetics PRS - Rhinitis	Waage *et al.* (2018)	8
Genetics PRS - Eczema	Paternoster *et al.* (2015)	425
Genetics PRS - Allergy	Ferreira *et al.* (2017)	4813
Genetics PRS - IgE sensitization	Bønnelykke *et al.* (2013)	221
Blood DNA methylation	Xu *et al.* (2018); Sarah *et al.* (2019); Zhang *et al.* (2019); Xu *et al.* (2021)	219
Nasal DNA methylation	Qi *et al.* (2020)	134

Caption

We used features that were found to be associated with allergic diseases in earlier studies. Especially for the multi-omics layers, a substantial filtering of features (SNPs and CpG sites) was needed to use these sources in ML models.

For the PRS, selected number of SNPs is based on GWAS significance threshold of 1e-07.

Supplementary References

1. Ordovas-Montanes, J. *et al.* Allergic inflammatory memory in human respiratory epithelial progenitor cells. *Nature* **560**, 649–654 (2018).
2. Wijga, A. H. *et al.* Cohort profile: the prevention and incidence of asthma and mite allergy (PIAMA) birth cohort. *Int. J. Epidemiol.* **43**, 527–535 (2014).
3. Pinart, M. *et al.* Comorbidity of eczema, rhinitis, and asthma in IgE-sensitised and non-IgE-sensitised children in MeDALL: a population-based cohort study. *Lancet Respir. Med.* **2**, 131–140 (2014).
4. Aryee, M. J. *et al.* Minfi: a flexible and comprehensive Bioconductor package for the analysis of Infinium DNA methylation microarrays. *Bioinformatics* **30**, 1363–1369 (2014).
5. Chen, Y. *et al.* Discovery of cross-reactive probes and polymorphic CpGs in the Illumina Infinium HumanMethylation450 microarray. *Epigenetics* **8**, 203–209 (2013).
6. Pidsley, R. *et al.* A data-driven approach to preprocessing Illumina 450K methylation array data. *BMC Genomics* **14**, 293 (2013).
7. Kim, D., Langmead, B. & Salzberg, S. L. HISAT: a fast spliced aligner with low memory requirements. *Nat. Methods* **12**, 357–360 (2015).
8. Li, H. *et al.* The Sequence Alignment/Map format and SAMtools. *Bioinforma. Oxf. Engl.* **25**, 2078–2079 (2009).
9. Anders, S., Pyl, P. T. & Huber, W. HTSeq--a Python framework to work with high-throughput sequencing data. *Bioinforma. Oxf. Engl.* **31**, 166–169 (2015).
10. Ritchie, M. E. *et al.* limma powers differential expression analyses for RNA-sequencing and microarray studies. *Nucleic Acids Res.* **43**, e47 (2015).
11. Forno, E. *et al.* DNA methylation in nasal epithelium, atopy, and atopic asthma in children: a genome-wide study. *Lancet Respir. Med.* **7**, 336–346 (2019).
12. Kim, S. *et al.* SNPs identified by GWAS affect asthma risk through DNA methylation and expression of cis-genes in airway epithelium. *Eur. Respir. J.* **55**, (2020).
13. Bisgaard, H. *et al.* Deep phenotyping of the unselected COPSAC $_{2010}$ birth cohort study. *Clin. Exp. Allergy* **43**, 1384–1394 (2013).
14. Blanken, M. O. *et al.* Respiratory syncytial virus and recurrent wheeze in healthy preterm infants. *N. Engl. J. Med.* **368**, 1791–1799 (2013).

15. Bisgaard, H. *et al.* Fish Oil–Derived Fatty Acids in Pregnancy and Wheeze and Asthma in Offspring. *N. Engl. J. Med.* **375**, 2530–2539 (2016).
16. Hanifin, J. M. & Rajka, G. Acta Derm Venereol (Stockh). (1980).
17. Morin, A. *et al.* Epigenetic landscape links upper airway microbiota in infancy with allergic rhinitis at 6 years of age. *J. Allergy Clin. Immunol.* **146**, 1358–1366 (2020).
18. Scheltema, N. M. *et al.* Respiratory syncytial virus prevention and asthma in healthy preterm infants: a randomised controlled trial. *Lancet Respir. Med.* **6**, 257–264 (2018).
19. Tingley, D., Yamamoto, T., Hirose, K., Keele, L. & Imai, K. Mediation: R package for causal mediation analysis. (2014).
20. Ferreira, M. A. *et al.* Shared genetic origin of asthma, hay fever and eczema elucidates allergic disease biology. *Nat. Genet.* **49**, 1752–1757 (2017).
21. Langfelder, P. & Horvath, S. WGCNA: an R package for weighted correlation network analysis. *BMC Bioinformatics* **9**, 559 (2008).
22. Breitling, R., Armengaud, P., Amtmann, A. & Herzyk, P. Rank products: a simple, yet powerful, new method to detect differentially regulated genes in replicated microarray experiments. *FEBS Lett.* **573**, 83–92 (2004).
