# Supplementary material for: Nasal DNA methylation at three CpG sites predicts childhood allergic disease
Source: Nat Commun. 2022 Dec 1;13:7415. doi: 10.1038/s41467-022-35088-6 (PMC9715628; doi:10.1038/s41467-022-35088-6)
Supplement: Supplementary file 3 — Description of Additional Supplementary Files [file 41467_2022_35088_MOESM3_ESM.pdf]

## Description of Additional Supplementary Files

**Supplementary Data 1**. eQTM analysis is performed using linear regression. Adjustment for multiple comparison is reflected in the use of false discovery rate (fdr)

**Supplementary Data 2**. SNP annotations are in the form <chromosome>.<position> and come from genome build hg19
