# Supplementary material for: Nasal DNA methylation at three CpG sites predicts childhood allergic disease
Source: Nat Commun. 2022 Dec 1;13:7415. doi: 10.1038/s41467-022-35088-6 (PMC9715628; doi:10.1038/s41467-022-35088-6)
Supplement: Supplementary file 6 — Reporting Summary [file 41467_2022_35088_MOESM6_ESM.pdf]

nature research

Corresponding author(s): Dr. Cheng-Jian Xu

Last updated by author(s): 14/02/22

Reporting Summary

Nature Research wishes to improve the reproducibility of the work that we publish. This form provides structure for consistency and transparency in reporting. For further information on Nature Research policies, see our Editorial Policies and the Editorial Policy Checklist.

Statistics

For all statistical analyses, confirm that the following items are present in the figure legend, table legend, main text, or Methods section.

n/a	Confirmed	
☐	☒	The exact sample size (*n*) for each experimental group/condition, given as a discrete number and unit of measurement
☐	☒	A statement on whether measurements were taken from distinct samples or whether the same sample was measured repeatedly
☐	☒	The statistical test(s) used AND whether they are one- or two-sided<br>*Only common tests should be described solely by name; describe more complex techniques in the Methods section.*
☐	☒	A description of all covariates tested
☒	☐	A description of any assumptions or corrections, such as tests of normality and adjustment for multiple comparisons
☐	☒	A full description of the statistical parameters including central tendency (e.g. means) or other basic estimates (e.g. regression coefficient) AND variation (e.g. standard deviation) or associated estimates of uncertainty (e.g. confidence intervals)
☐	☒	For null hypothesis testing, the test statistic (e.g. *F*, *t*, *r*) with confidence intervals, effect sizes, degrees of freedom and *P* value noted<br>*Give P values as exact values whenever suitable.*
☒	☐	For Bayesian analysis, information on the choice of priors and Markov chain Monte Carlo settings
☒	☐	For hierarchical and complex designs, identification of the appropriate level for tests and full reporting of outcomes
☐	☒	Estimates of effect sizes (e.g. Cohen's *d*, Pearson's *r*), indicating how they were calculated

Our web collection on statistics for biologists contains articles on many of the points above.

Software and code

Policy information about availability of computer code

Data collection

Genotyping was performed in four phases using four different platforms, being Illumina Human610 quad array, Illumina HumanOmniExpress array, Illumina Human Omni Express Exome Array, and Illumina Infinium Global Screening Array. Imputation of the Quality control on the genotype sequencing data was performed using plink (1.07). Imputation was done on the Michigan imputation server (v1.2.4). VCFtools (0.1.12b) is used to get the SNP dosages.

Blood and nasal DNA methylation were measured by Infinium HumanMethylation450 BeadChip array. The minfi (1.24.0) in R (3.5.1) package was used to perform quality control and preprocessing.

After sequencing of the RNA data, HISAT (version 0.1.5) was used to align to b37; SAMtools (version 1.2) was used to sort the aligned reads. Gene level quantification was performed by HTSeq (version 0.6.1p1). Quality control metrics were calculated for the raw sequencing data using the FastQC tool (version 0.11.3) and QC metrics were calculated for the aligned reads using Picard-tools (version 1.130).

Ambient RNA was corrected using FastCAR, and Scrublet64 was used for identifying doublets.

See supplementary methods for extensive explanation on sequencing procedure.

Data analysis

The prediction model is produced using the open source R (3.5.1) software. Package caret (6.0--86) was primarily used for model training and evaluation.
Our trained allergy prediction model and its code are freely available at https://github.com/GRIAC-Bioinformatics/Allergy_prediction. The model is ready to be used for predicting allergic disease with the three CpG sites as the input. Code for retraining the model on new datasets is also provided.

Methylation count data were transformed to log2CPM and analyzed using the voom function in the limma (3.34.9) package in R. KEGG pathway enrichment analysis was performed using R package topGO, R package Seurat (4.0) was used for downstream analysis of single-cell RNA seq. Harmony was used to integrate data from different cohorts. MatrixEQTL67 in R was used to perform the MeQTL analysis and the Mediation package facilitated the mediation analysis.

For manuscripts utilizing custom algorithms or software that are central to the research but not yet described in published literature, software must be made available to editors and reviewers. We strongly encourage code deposition in a community repository (e.g. GitHub). See the Nature Research guidelines for submitting code & software for further information.

Data

Policy information about availability of data

All manuscripts must include a data availability statement. This statement should provide the following information, where applicable:

- Accession codes, unique identifiers, or web links for publicly available datasets
- A list of figures that have associated raw data
- A description of any restrictions on data availability

Nasal and blood DNA methylation data from the discovery cohort (PIAMA) have been deposited in the European Genome-phenome Archive (EGA), which is hosted by the European Bioinformatics Institute (EMBL-EBI) and the Centre for Genomic Regulation (CRG), under accession number EGAS00001005189, dataset EGAD00010002263. Raw data to generate figures and tables are available from the corresponding author with the appropriate permission from the PIAMA study team and investigators upon reasonable request and institutional review board approval. The GWAS summary statistics used for the PRS can be found in the public GWAS catalog under the following links: allergy (https://www.ebi.ac.uk/gwas/publications/29083406); asthma (https://www.ebi.ac.uk/gwas/publications/29273806); rhinitis (https://www.ebi.ac.uk/gwas/publications/30013184); eczema (https://www.ebi.ac.uk/gwas/publications/26482879); sensitization (https://www.ebi.ac.uk/gwas/publications/23817571). For the PRS analysis, human genome build GRCh37 was used, while for the single-cell RNA analysis GRCh38 1.2.0 was used. Single-cell RNAseq data for Supplementary Figure 9 is from Ordovas-Montanes et al. (2018) and publicly available (https://www.nature.com/articles/s41586?018?0449?8).

Field-specific reporting

Please select the one below that is the best fit for your research. If you are not sure, read the appropriate sections before making your selection.

☒ Life sciences ☐ Behavioural & social sciences ☐ Ecological, evolutionary & environmental sciences

For a reference copy of the document with all sections, see nature.com/documents/nr-reporting-summary-flat.pdf

Life sciences study design

All studies must disclose on these points even when the disclosure is negative.

Sample size	Study design of the PIAMA cohort has been previously published by Wijga, A.H. et al. (2014). Sample size in this study is based on complete data availability for subjects for all relevenat data layers (genetics, blood and nasal DNA methylation, environment, perinatal, host) in the PIAMA dataset.<br><br>Wijga, A. H. et al. Cohort profile: the prevention and incidence of asthma and mite allergy (PIAMA) birth cohort. Int J Epidemiol 43, 527–535 (2014).
Data exclusions	All subjects with complete data were used by the authors.<br>For genotype data, following QC criteria are used: imputation quality score Rsq >0.8; MAF>0.01 and HWE <1*10-12.<br>For methylation and RNA-seq data, QC steps have been published previously, see Qi, C. et al. (2029)<br><br>Qi, C. et al. Nasal DNA methylation profiling of asthma and rhinitis. J. Allergy Clin. Immunol. (2020) doi:10.1016/j.jaci.2019.12.911.
Replication	The prediction model was replicated in three independent cohorts. We used the Epigenetic Variation and Childhood Asthma study in Puerto Ricans study (EVA-PR, including subjects aged 9 to 20 years), as well as two cohorts of younger children (mean age 6 years) COPSAC2010 and and MAKI. Details of these cohorts are shown in the supplementary materials.
Randomization	Sample plates and chips were fully randomized to ensure to minimize potential confounding by batch effects.<br>For the development of the machine learning model, subjects were randomly split over the various fold in the cross-validation framework.
Blinding	No blinding was applied as this study was performed on the PIAMA cohort, which was an observational human study.

Reporting for specific materials, systems and methods

We require information from authors about some types of materials, experimental systems and methods used in many studies. Here, indicate whether each material, system or method listed is relevant to your study. If you are not sure if a list item applies to your research, read the appropriate section before selecting a response.

Materials & experimental systems

n/a	Involved in the study
x	Antibodies
x	Eukaryotic cell lines
x	Palaeontology and archaeology
x	Animals and other organisms
	x Human research participants
x	Clinical data
x	Dual use research of concern

Methods

n/a	Involved in the study
x	ChIP-seq
x	Flow cytometry
x	MRI-based neuroimaging

Human research participants

Policy information about studies involving human research participants

Population characteristics	For this study, 348 participants of the PIAMA cohort were used, being 16 years of age with 51.3% being male. Allergy phenotype was registered in 19.3% of the population, while 46.6% has IgE sensitisation.
Recruitment	The complete recruitment procedure of the PIAMA cohort has been described in Wijga, A.H. et al. (2014).<br>Pregnant mothers were selected from the general population and neligible selection bias is expected for participation. While participation rate in follow-ups is high, this could include a potential risk of self-selection bias. Based on consultation with original authors/creators of the PIAMA cohort, there is no indication that this would heavily effect model results.<br><br>Wijga, A. H. et al. Cohort profile: the prevention and incidence of asthma and mite allergy (PIAMA) birth cohort. Int J Epidemiol 43, 527–535 (2014).
Ethics oversight	The Medical Ethical Committees of the participating institutes approved the study (Utrecht and Groningen METC (Medisch Ethische Toetsings Commissie) protocol number 12-019/K).

Note that full information on the approval of the study protocol must also be provided in the manuscript.
